# Construction of a highly saturated linkage map in Japanese plum (*Prunus salicina* L.) using GBS for SNP marker calling

**DOI:** 10.1371/journal.pone.0208032

**Published:** 2018-12-03

**Authors:** Basilio Carrasco, Máximo González, Marlene Gebauer, Rolando García-González, Jonathan Maldonado, Herman Silva

**Affiliations:** 1 Pontificia Universidad Católica de Chile, Facultad de Agronomía e Ingeniería Forestal, Departamento de Ciencias Vegetales, Macul, Santiago, Chile; 2 Sociedad *Bio*TECNOS Ltda, R&D Department Camino a Pangal Km 2 1/2, San Javier, Región del Maule, Chile; 3 Facultad de Ciencias Agrarias y Forestales, Centro de Biotecnología de los Recursos Naturales (CENBio), Universidad Católica del Maule, Talca, Chile; 4 Universidad de Chile, Facultad de Ciencias Agronómicas, Departamento de Producción Agrícola, Laboratorio de Genómica Funcional & Bioinformática, La Pintana, Santiago, Chile; ICAR-Indian Institute of Agricultural Biotechnology, INDIA

## Abstract

This study reports the construction of high density linkage maps of Japanese plum (*Prunus salicina* Lindl.) using single nucleotide polymorphism markers (SNPs), obtained with a GBS strategy. The mapping population (An x Au) was obtained by crossing cv. “Angeleno” (An) as maternal line and cv. “Aurora” (Au) as the pollen donor. A total of 49,826 SNPs were identified using the peach genome V2.1 as a reference. Then a stringent filtering was carried out, which revealed 1,441 high quality SNPs in 137 An x Au offspring, which were mapped in eight linkage groups. Finally, the consensus map was built using 732 SNPs which spanned 617 cM with an average of 0.96 cM between adjacent markers. The majority of the SNPs were distributed in the intragenic region in all the linkage groups. Considering all linkage groups together, 85.6% of the SNPs were located in intragenic regions and only 14.4% were located in intergenic regions. The genetic linkage analysis was able to co-localize two to three SNPs over 37 putative orthologous genes in eight linkage groups in the Japanese plum map. These results indicate a high level of synteny and collinearity between Japanese plum and peach genomes.

## Introduction

The genus *Prunus* (family *Rosaceae*) contains more than 200 species, which include some relevant agricultural stone fruit crops such as peach, apricot, cherry, Japanese plum, myrobalan and European plum, among others [1, 2]. Japanese plum is a self-incompatible, diploid species (2n = 2X = 16) that produces an edible drupe; it has been cultivated for 4,000 years [2] for fruit production and ornamental purposes. Different centers around the world have begun to perform breeding programs to obtain new cultivars. Usually the new cultivars show a high level of variability for some important traits such as harvest date, fruit size, flesh color and shape [3]. Some genetic studies have been carried out to investigate the inheritance pattern and heritability of flowering date, ripening date, fruit size, higher total soluble solids, sweetness and flavor in the fruit [2, 3], while molecular studies have been few and focused only on the ethylene response and skin and flesh colors [4–10]. The construction of a linkage map is an important tool for genetic studies. This map will allow establishing the organization of a genome as well as a relationship between markers and polymorphisms. The mapping information offers evidence about polymorphisms strongly associated with agronomically relevant traits in segregating populations [11, 12], allowing the genetic basis of the trait of interest to be determined, and also can be a powerful tool in breeding programs for choosing parental lines and implementing marker-assisted selection strategies. The availability of a consensus reference map built for Rosaceae has facilitated the order and the physical orientation of maps as well as the localization of new consensus molecular markers for assisted selection in different *Prunus* species. Several genetic maps have been published for Rosaceae species, including peach, apple, pear, raspberry, and cherry. A comparative linkage map has even been built for Rosaceae [13, 14]. Peach has been considered as a model species for the genus *Prunus* [15, 16]. Verde et al. [17] recently published a new version of a high-quality draft genome of peach, which is a baseline for comparative analysis with *Prunus* species. In addition, Zhang et al. [18] have published the genome of mei (*Prunus mume*).

However, to date only a few genetic linkage maps have been published for plums and related species. Dirlewanger et al. [13] developed the first genetic map for mirobalan plum (*P*. *ceracifera*). Later, 144 microsatellites (or simple sequence repeats, SSR) were mapped in *Prunus mume* [19]. Only two genetic maps have been published for *Prunus salicina*. Vieira et al. [20] built a map using AFLPs and more recently, Salazar et al. [21] described QTLs using SNPs.

Next generation sequencing approaches have recently allowed the release of whole genome reference sequences for many plant species. This technique also allows the identification of thousands of single nucleotide polymorphisms (SNPs), the most abundant type of DNA marker found in eukaryotic genomes [22–24]. SNPs have become tremendously important as markers for genetics research in plants because they have been found to be in high frequency, display a lower mutation rate compared to SSR-based markers and they are uniformly distributed across the genome [25].

The distribution pattern of SNPs in segregating populations plus the available reference genomes have allowed analyzing the linkage relationships and the physical genome distribution of those SNPs, permitting comparisons of the collinearity between related species [26]. These properties make SNPs relevant to carry out genetic studies such as phylogenetics, genetic diversity, association analysis and genetic mapping [27].

Next generation sequencing is rapidly becoming a low-cost technology to carry out massive genetic studies, which is allowing important advances in plant genetics and breeding. Elshire et al. [28] developed a robust and low-cost genotyping method based on partial genome sequencing called genotyping-by-sequencing (GBS). This approach uses restriction enzymes to digest the genome in order to reduce its complexity. The DNA fragments are sequenced by high-throughput methods, obtaining hundreds of thousands of SNPs simultaneously. The GBS approach has been shown to be suited to do genetic analysis and linkage mapping of *Rosaceae* species such as peach [29], sweet cherry [30, 31], raspberry [32], and apple [33]. The objective of the present study was the development of a high-density linkage map which will provide important information about the genetics and genomics of *Prunus salicina* and its relationships with other *Prunus* species. To date this is the most saturated map available for this species.

## Material and methods

### Parental lines and F_1_ segregating population

During the spring of 2013 we crossed the cultivar “Angeleno” (An) (late harvest variety; 179 days after full blossom) as a maternal line with the cultivar “Aurora” (Au) (early harvest variety; 112 days after full blossom) as the pollen donor. An was selected as a parental line because is the most important cultivar of Japanese plum for the Chilean fresh fruit exportation industry [34]. An is a late harvest cultivar and has a high level of productivity and a long period of shelf life. Its fruits can be stored at 4 °C for a long period of time (at least 45 days) without expressing physiological disorders such as woolliness or internal breakdown [35]. To obtain seedlings after pollination and then fruit set, the seed were collected and germinated at 4 °C for 75 days. Then 137 F_1_ seedlings were established at the experimental station of the Fundación Agro UC located at Curacaví, Chile (33° 26’ South, 71° 01’ West) during September, 2014. Each seedling was planted directly in the field without grafting and maintained following standard agronomic protocols (canopy pruning, fruit thinning, drip irrigation, fertilization and phytosanitary control).

### Genotyping by sequencing (GBS)

GBS protocols were carried out by Institute of Biotechnology, Cornell University, Ithaca, NY, USA according to [28] (more details can be obtained from http://www.biotech.cornell.edu/brc/genomics-facility). Young leaves from the 137 segregating F_1_ and parental lines An and Au were collected for DNA isolation. High quality DNA was extracted from leaves using a standard CTAB approach modified by Carrasco et al. [36]. The double-stranded DNA concentrations were measured with a Qubit 3.0 Fluorometer (Thermo Fisher Scientific). We extracted at least 70 ng/ul of double-stranded DNA per sample according to the protocol suggested by the BRC Genome Facility (Cornell University Biotechnology Resource Center, USA). GBS libraries were developed using the restriction enzyme ApeKI (GCWGC) and two different adapters according to protocols from the Institute for Genome Diversity (IGD) at Cornell University. ApeKI was selected from a panel of restriction enzymes because it is able to produce hundreds of thousands of fragments between 150 and 500bp. Several studies of the genomes of *Prunus* species have used this criterion successfully to select the restriction enzyme for GBS [21; 28; 31; 37; 38]. GBS sequencing libraries were prepared by ligating the digested DNA to nucleotide adapters (barcodes), followed by standard PCR. Sequencing was performed using Illumina HiSeq2000. The DNA of the parental lines was sequenced three times (independent samples) in order to reduce missing data and errors during SNP calling.

The separate FASTQ (raw files) were aligned to Peach v2.1 [17] (http://www.rosaceae.org/gb/gbrowse/prunus_persica_v2.1) using the Burrows-Wheelers alignment tool version 0.7.8-r441 [39]. Alignments were converted to the SAM format, then merged and sorted into one master binary alignment file with SAMtools 0.1.18 [39]. A ‘master’ TagCounts file was produced, which was aligned to the peach genome V2.1, and a Tags on Physical Map (TOPM) file was built, containing the best genomic position of each tag. The barcode information in the original FASTQ files is used to tally the number of times each tag in the master tag list is observed in each sample (‘taxon’) and these counts are stored in a TagsByTaxa file. The information recorded in the TOPM and TBT is then used to discover SNPs and filter them based upon the proportion of taxa covered by the TagLocus, minor allele frequency and inbreeding coefficient (F_IT_). More details about the pipeline to SNP calling can be found in Glaubitz et al. [40] and IGD.

Finally, the identified SNPs corresponding to each genotype were stored as a filtered VCF file for posterior filtering with Tassel 5.2.16 [40, 41] and mapping analysis using Joinmap v4.1 software [42].

### SNP analysis

SNPs were identified following the nomenclature defined in the peach genome v2.1, where each SNP was located in the pseudomolecules (scaffold) s1 to s8, followed by the physical position in base pairs (bp). Total SNPs were filtered using the VCFtools software [43]. Only biallelic SNPs with minimum allele frequency of 0.05 were used. SNP markers with more than 10% missing data were removed, and only those SNPs that showed an even distribution along the main eight peach scaffolds were used for posterior analysis (high quality SNPs). The location of each SNP within intergenic and genic regions (exon, intron and untranslated regions (UTR) was determined using the Perl script (www.perl.org), determining the positions from the *Prunus persica* v2.1 GFF annotation.

### Linkage map construction

The VCF file of clean SNPs was converted to JoinMap format using ngsTools software [44]; therefore the SNPs that were classified as heterozygous in one of the parents were scored as segregation types <lmxll> or <nnxnp>, while SNPs heterozygous in both parents were scored as segregation type <hkxhk>.

Map construction was performed using JoinMap 4.1 software [42] following a two-step strategy. Parental maps were first constructed using SNPs classified as <lmxll> or <nnxnp>. Then for consensus map construction, the integration of the parental maps was performed including the SNPs classified as <hkxhk>.

The segregation distortion of SNPs was determined by calculating chi-square (χ^2^) using JoinMap, and SNPs with a major distortion of p < 0.001 were removed. In addition, in those cases in which one SNP had *Similarity of Loci* = 1 with another SNP, only one of them was used for map construction.

Maternal, paternal and consensus map constructions were performed using the regression mapping algorithm. For maternal and paternal map construction, markers were grouped using a minimum independence LOD (logarithm of the odds) score of 10.0 and linkage groups were established at a minimum LOD score of 3.0 and maximum recombination frequency of 0.40. Map distance was estimated using the Kosambi mapping function [45]. Consensus map construction was performed considering the same parameters used for parental map constructions.

## Results

### Linkage mapping

Using the GBS approach we obtained 454,912,276 reads and 4,995,919 tags; 2,367,021 tags (47.4%) were aligned to a unique position, 200,809 tags (4.0%) were aligned to multiple positions and 2,428,089 tags (48.6%) could not be aligned to the peach genome v2.1 that was used as a reference. The HapMap file containing 49,826 filtered SNPs was used for subsequent analysis. After applying filters (MAF >0.05 and 10% as maximum missing data) a total of 12,720 SNPs were obtained to be analyzed with the software JoinMap 4.1. Chi-square analyses were performed on informative SNPs to evaluate their conformity to the expected Mendelian segregation ratios. SNPs with identical segregation patterns were also discarded. Finally, 1,441 high quality SNPs were available for map construction.

Of the 1,441 high quality SNPs, 714 (50%) SNPs were classified as female parent segregation (“Angeleno”; <nn x np>); 320 (22%) as male parent segregation (“Aurora”; <lm x ll>) and 407 (28%) SNPs as heterozygous segregation in both parents (<hk x hk>).

**Fig 1:**
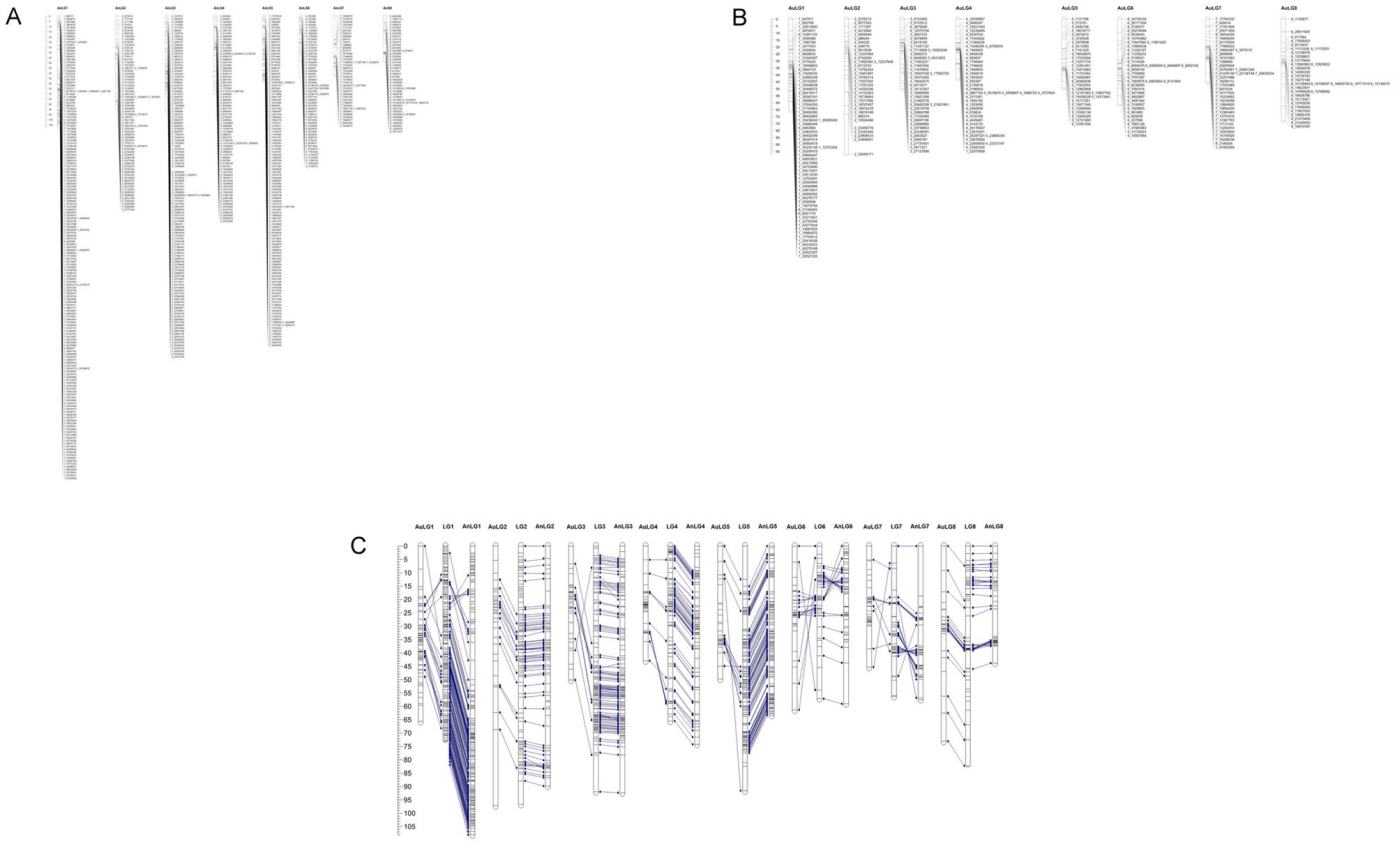
Genetic map. a. “Angeleno” (female line). b. “Aurora” (male line). c. Consensus.

The linkage analysis showed that the 1,441 SNPs were spread over eight linkage groups, which corresponds to the reported haploid chromosome number in Japanese plum [2]. The linkage maps spanned 588 cM and 490 cM for An (female line) and Au (male line), respectively (Fig 1a and 1b; S1 Table).

The consensus linkage map was constructed using SNPs mapped in both parental maps plus the SNPs in heterozygous configuration. The consensus map contained 732 non-redundant SNPs and spanned a distance of 617 cM (Table 1; Fig 1c; S1 and S2 Tables). The number of SNPs mapped, the total map length, the average distance and maximum gap (Table 1) ranged from 47 SNPs, 56 cM, 1.22 cM/SNP and 8.0 cM in LG7 to 89 SNPs, 100cM, 1.13 cM/ SNP and 10.2 cM for LG2. The average distance between markers was 0.96 cM/SNP.

**Table 1:** Characterization of 732 SNPs distributed in the consensus genetic map of Japanese plum.

Linkage Group	SNPs (N°)	Total distance (cM)	Average distance (cM/SNP)	Maximum gap (cM)
LG1	161	73	0.45	5.3
LG2	89	100	1.13	10.2
LG3	125	92	0.74	13.8
LG4	82	66	0.81	4.3
LG5	121	92	0.76	12.4
LG6	48	57	1.21	8.4
LG7	47	56	1.22	8.0
LG8	59	82	1.42	9.7
**Total**	**732**	**617**	**0.96**	**9.0**

Most of the SNPs identified using the peach genome were co-localized in the correct linkage group after linkage analysis. However, some SNPs did not follow the same physical order relative to the scaffolds of peach genome v2.1. We found that 69 SNPs (9.5%) of 732 SNPs mapped to different linkage groups compared to the peach genetic map.

### SNP distribution through the linkage groups

**Fig 2:**
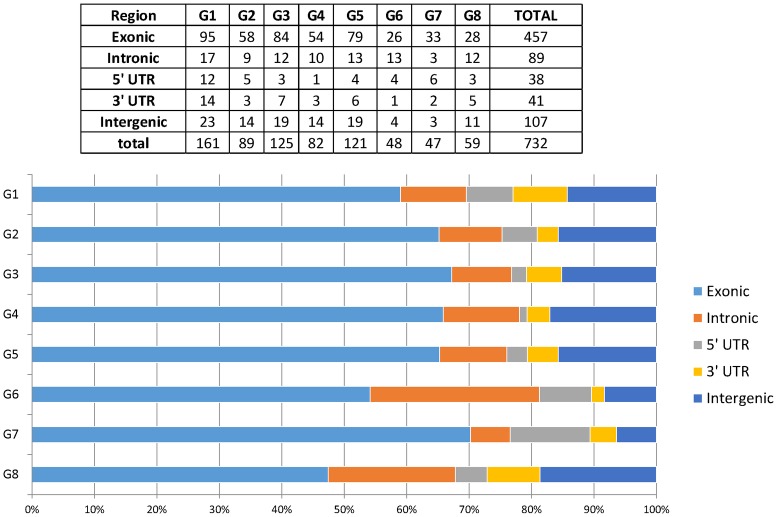
Genomic distribution of 732 SNPs in *P*. *salicina* based on physical positions identified in peach genome v2.1.

The location of each SNP in the intragenic and intergenic regions (Fig 2) was determined by using the physical position in peach genome v2.1. Table 2 shows the distribution of SNPs in the intragenic as well as intergenic regions of the eight linkage groups.

**Table 2:** Distribution of SNPs in linkage groups.

Linkage Group (LG)	SNPs in intragenic regions (%)	SNPs in intergenic regions (%)
LG1	85.7	14.3
LG2	85.2	14.8
LG3	85.5	15.2
LG4	82.9	17.1
LG5	84.3	15.7
LG6	91.7	8.3
LG7	93.6	6.4
LG8	81.4	18.6

Considering all linkage groups together, 85.6% of the SNPs (625 SNPs) were located in intragenic regions (62.6% in exons; 12.2% in introns and 10.8% in UTR), and only 14.4% (107 SNPs) were located in intergenic regions (Fig 2).

**Fig 3:**
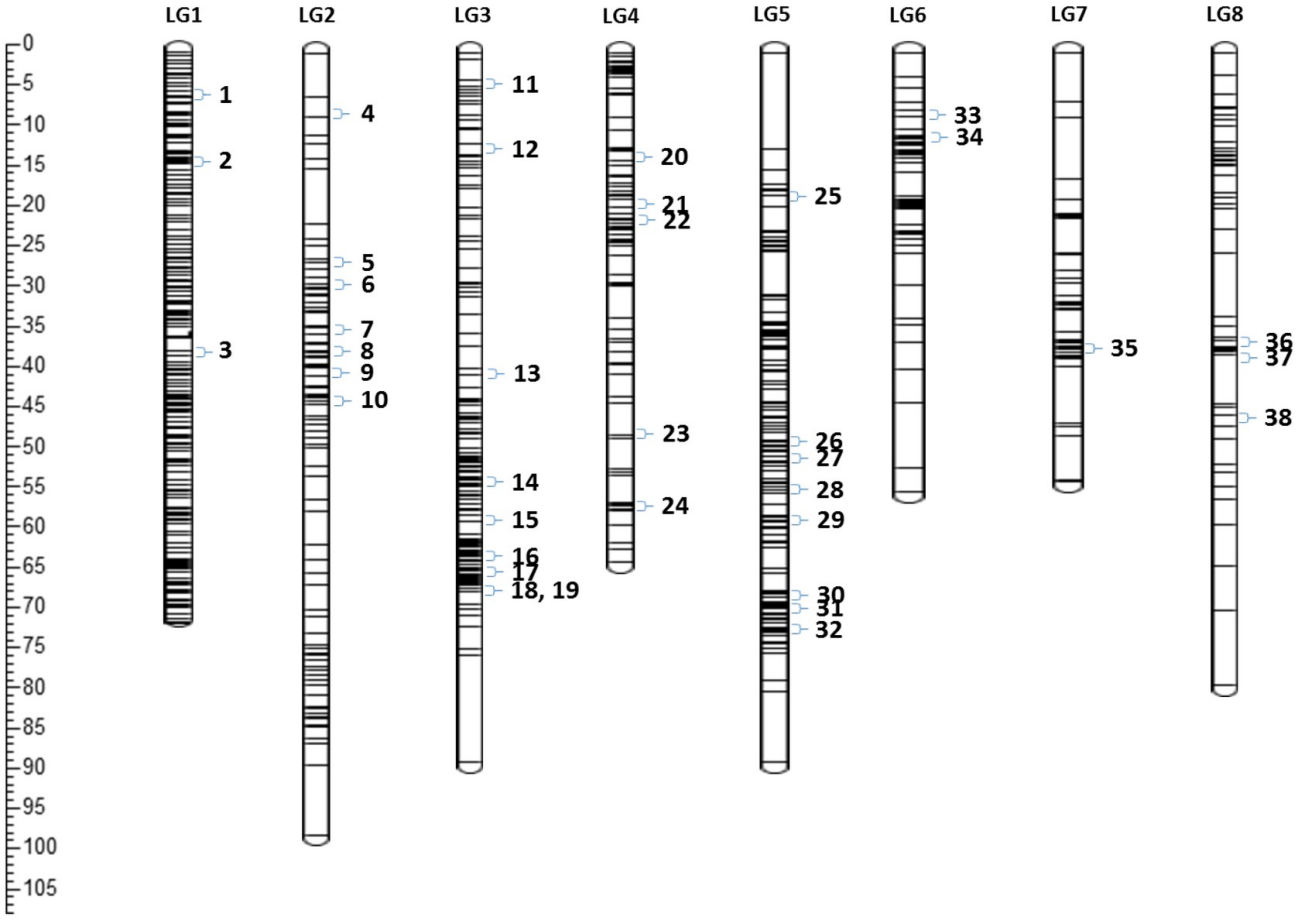
Japanese plum consensus genetic map. The numbers indicate the map position of 38 putative orthologous genes in eight linkage groups of Japanese plum (see supplementary material for details). These genes were identified according to the annotation of peach genome v2.1.

The genetic linkage analysis was also able to co-localize at least 1 SNP in 460 putative orthologous genes identified in the peach genome. These genes were distributed at the linkage groups as follows: LG1 = 112; LG2 = 50; LG3 = 65; LG4 = 52; LG5 = 79; LG6 = 34; LG7 = 34 and LG8 = 34 (for more details about these SNPs and genes see S2 Table). Using linkage analysis we were able to co-localize two to three SNPs simultaneously in 38 genes in the eight linkage groups of the genetic map of Japanese plum (Fig 3).

## Discussion

Genetic linkage maps are used as a tool for primary localization of important genomic regions associated with the genetic control of both qualitative and quantitative traits, which can help to support a breeding program [46]. The huge amount of genetic information that can be obtained from high throughput sequencing approaches today have allowed whole-genome sequencing and linkage analysis to become powerful methodologies for the identification of genetic polymorphisms related to complex traits in different species [47, 48]. GBS is able to generate abundant SNPs for a large number of individuals of a species with a relatively low cost per sample [49]. It is also an effective source of sequence tags that can be used as genetic anchors to direct contig/scaffold assembly and to map genomic fragments using a reference genome.

The GBS approach used in this study identified 49,826 SNPs to initiate the mapping analysis. However, most of them (97.1%) were not used for the construction of a linkage genetic map and were eliminated due to low minor allele frequency (<0.05), excess of missing data (>10%), SNPs with incorrect genotype, more than two alleles for one position and distortion of segregation. It is important to highlight that according to Bastien et al., [50] the restriction enzyme ApeKI used to generate the genomic library in GBS approach [28] helps to identify more SNPs than other restriction enzymes, but with a lower read coverage per marker and resulting in more missing data. After applying the filtering criteria, 1,441 high quality SNPs were available to build the genetic map in Japanese plum. Those 1,441 SNPs represented only 2.9% of the total SNPs identified, which shows the limitations of the GBS approach. Davey et al., [49] and Jiang et al., [51] suggested that the main limitation of GBS is the presence of missing data due to low coverage genotyping, inconsistency in the number of sites sequenced per sample and number of reads per site.

New methodologies to discover SNPs that can bypass these difficulties could give a more consistent number of useful SNPs for carrying out genetic studies [51–53]. In contrast to traditional GBS [28], Specific-Locus Amplified Fragment Sequencing (SLAF-seq) [51–53] is an new and efficient method for SNP discovery and genotyping that does not require a reference genome, uses two restriction enzymes (HaeIII and MseI), selecting fragments between 370-450bp. SLAF-seq is able to produce a larger number of high quality SNPs for mapping purposes [53].

Linkage maps using SNPs have been published for several species of the Rosaceae, including raspberry [32], apple [33, 54], pear [55], peach [29, 56], sweet cherry [30, 31], Chinese plum [57], Japanese plum [21] and almond [37]. These linkage maps were able to identify thousands of molecular markers; nevertheless, after filtering only a reduced number of SNPs were used to build a genetic map.

Few genetic linkage maps have been published for plum species. The first genetic map for plum was published by Dirlerwanger et al. [13]. They reported a map of a myrobalan clone (*P*. *ceracifera*) using 93 markers (two SCARs plus 91 SSR) which expanded over eight linkage groups, covering 524.8 cM. Later, Vieira et al. [20] reported a linkage map for parental lines of Japanese plum using 56 to 84 AFLP which covered 905.5 to 1,349.6 cM with an average distance between markers of 16.1 cM to 16.2 cM.

Recently Salazar et al., [21] published a genetic map using SNPs obtained by GBS. They mapped a total of 981 SNPs, 479 SNPs for the female line and 502 SNPs for the male line (cv. “Angeleno”), covering 688.8 cM and 647.03 cM, respectively. The average distance between SNPs was two cM. However, Salazar et al., [21] did not report a consensus map for the two parental lines, therefore it is not possible to distinguish redundant SNPs.

In contrast, we analyzed 1,441 high quality SNPs. Of these, 714 SNPs were mapped in the female parent (cv. “Angeleno”) and 320 in the male parent (cv. “Aurora”) expanded over 578cM and 472 cM, respectively. Our consensus map was built using 732 non-redundant SNPs, covering 617 cM with an average distance between SNPs of 0.96 cM. This saturation represents at least twice that reported by Salazar et al. [21] and is considered a highly saturated map according to Slate et al. [58]. These authors indicated that a high-density map refers an average interval between SNPs less than two cM. It is important to note that both our study and that of Salazar et al., [21] used the “Angeleno” cultivar as a parental line, which could be favorable for future studies.

Comparing our linkage map with those previously built for Rosaceae species (Table 2), the map length and density of the SNP were similar to those published for *P*. *persica* [56] and *P*. *avium* [30, 31]. Up to now only the *P*. *mume* map has higher saturation, with a density of 0.15 to 0.24 cM/SNP [57]. However, the *P*. *mume* map has large gaps in LG1 (24.13cM); LG6 (12.79cM) and LG8 (24.13cM). In contrast, the Japanese plum map reported in this study showed a maximum gap of 13.8cM, which was a little more than half that of *P*. *mume*. The presence of gaps in a linkage map needs to be analyzed carefully, because they could indicate an assembly problem in the reference genome. Plant genomes have proved to be difficult to assemble because of high heterozygosity, large numbers of repeat sequences and being large and complex [59]. Eichler et al., [60] suggested that genome duplication will be the most important cause of gaps in a physical and genetic map because they are difficult to assemble due to mapping of markers to multiple regions. Reduction of the gaps by increasing the number of offspring and molecular markers will improve the density and resolution of linkage maps, enhancing the ability to identify QTLs and markers for assisted selection.

Table 3 indicates that the density of our Japanese plum map (cM/SNPs) was rather superior to those obtained for previous maps developed for other species of Rosaceae such as raspberry, apple, and pear. The number of offspring (137) and the number of SNPs analyzed (730) in the segregating populations (An x Au) could explain this result.

**Table 3:** Coverage and density reported in available genetic maps for some Rosaceae species developed using SNPs.

Species	SNPs (Nr)	Map length (cM)	Density (cM/SNP)
*Prunus salicina* ^a^	730	626	0.96
*Prunus salicina* [21]	479–502	647.03–688.8	2.0
*Prunus pérsica* [49]	588	454	0.81
*Prunus avium* [30]	687–723	639.9–752.9	0.9–1.1
*Prunus avium* [31]	987	731.3	0.7
*Prunus mume* [50]	8,007	1,550.62	0.15–0.24
*Rubus ideaus* [32]	2,391–4,521	376.6–462.7	0.1–0.16
*Malus pumila* [51]	15,417	1,267	0.37
*Pyrus communis* [52]	3,853	3,266	0.64

^a^This study

The Japanese plum map revealed that most of the SNPs (90.5%) were located in the linkage groups according to their physical locations in the scaffold of the peach genome. However, some discrepancies were observed, such as differences in the correlative order of the SNPs in the genetic map and differences between physical locations of the SNPs in the peach genome and their positions in the linkage groups. Such discrepancies in SNP order have been previously reported in almond [61], sweet cherry [30, 31] and mei (*P*. *mume*) [57]. Also, some few SNPs (9.5%) located physically in the peach scaffold mapped to a different linkage group in the Japanese plum map. These discrepancies could be explained by assembly errors in the peach genome [30], mapping errors due to the number of individuals analyzed, and some evolutionary process such as genomic translocations and deletions.

Genus *Prunus* has shown conserved intraspecific and intragenic co-linearity in the Rosaceae [13], also even with other genera such as *Populus* [13]. Conserved synteny is important to plant breeding because it can be exploited to identify molecular markers linked to agronomically relevant traits between related species. A recent example of synteny-based development of molecular markers was done between peach and almond [62]. Therefore, based on the synteny pattern observed between *Prunus* species we tried to identify SNPs mapped in Japanese plum in genes localized in the V2.1 peach genome. We were able to find 510 putative orthologous genes by using SNPs from the peach genome as a reference. 38 genes out of the 510 putative orthologues were co-localized with two to three SNPs after genetic linkage analysis was carried out in our segregating F_1_ population of Japanese plum. Our result suggests synteny between the Japanese plum and peach genomes (See Fig 3).

We found that 85.4% of the SNPs analyzed were located in intragenic regions. Pootakham et al., [63] and Bastien et al., [50] suggested that methylation-sensitive enzymes such as ApeKI increase the degree of enrichment in coding regions of the genome. More than 50% of the SNPs were located within or close to genic regions.

The number of SNPs identified in our study was superior to those reported by Klagges et al. [30] and Guajardo et al. [31] in sweet cherry (See Fig 2). We have obtained a highly saturated linkage map for *P*. *salicina* (Japanese plum) that will be important in breeding studies as well as for the genome sequencing of this species being developed around the world (Pacheco, I. and Silva, H., unpublished data; Fernandez, A., unpublished data).

## Supporting information

S1 Table Genetic distance between SNPs mapped by linkage groups. The information is given for the female, the male and the consensus map according to peach genome version v2.1.
(DOCX)

S2 Table SNPs information related to the linkage map and position on the peach genome version v2.1. **Table A: Linkage map of female line cv ‘Angeleno’**. For each SNP is described his original linkage group, the position on the physical map and the position on the genetic map. Physical map position according to peach genome V2.1. **Table B**: **Linkage map of male line cv ‘Aurora’**. For each SNP is described its original linkage group, the position on the physical map and the position on the genetic map. Physical map position according to peach genome V2.1. **Table C: Consensus linkage map**. For each SNP is described its original linkage group, the position on the physical map, the position on the genetic map and corresponding orthologous genes that co-localize 2 to 3 SNPs simultaneously (n = 38). Physical map position according to peach genome V2.1. **Table D: Identification of the SNPs that co-localize 2 to 3 SNPs simultaneously over 38 genes**. Physical map position according to peach genome V2.1. **Table E: Functional annotation (Phytozome) of 38 putative orthologous genes that co-localize 2 to 3 SNPs simultaneously. Table F: SNPs identification over 460 putative orthologous genes without multiple SNP co-localization**. Physical map position according to peach genome V2.1.
(XLSX)
