# Supplementary material for: Construction of a highly saturated linkage map in Japanese plum (Prunus salicina L.) using GBS for SNP marker calling
Source: PLoS One. 2018 Dec 3;13(12):e0208032. doi: 10.1371/journal.pone.0208032 (PMC6277071; doi:10.1371/journal.pone.0208032)
Supplement: S1 Table — The information is given for the female, the male and the consensus map according to peach genome version v2.1. (DOCX) [file pone.0208032.s001.docx]

| Linkage group | Female parent (“Angeleno”) | Male parent (“Aurora”) | Consensus |
| --- | --- | --- | --- |
| 1 | 108.024 | 65.583 | 72.745 |
| 2 | 89.795 | 97.096 | 99.683 |
| 3 | 92.282 | 50.065 | 91.881 |
| 4 | 74.221 | 42.869 | 65.505 |
| 5 | 63.252 | 49.749 | 91.552 |
| 6 | 59.000 | 61.263 | 57.098 |
| 7 | 57.423 | 45.344 | 56.173 |
| 8 | 48.831 | 77.995 | 88.229 |
| Total | 587.828 | 489.964 | 622.836 |
